# Spectral tuning and after-effects in neural entrainment

**DOI:** 10.1186/s12993-024-00259-6

**Published:** 2024-11-21

**Authors:** Maëlan Q. Menétrey, David Pascucci

**Affiliations:** 1 Laboratory of Psychophysics, Brain Mind Institute, École Polytechnique Fédérale de Lausanne (EPFL), Lausanne, Switzerland; 2 Psychophysics and Neural Dynamics Lab, Department of Radiology, Lausanne University Hospital (CHUV) and University of Lausanne (UNIL), Lausanne, Switzerland; 3 The Sense Innovation and Research Center, Lausanne, Switzerland

**Keywords:** EEG, Neural entrainment, Evoked responses, Alpha oscillations, Long-lasting effects

## Abstract

Neural entrainment has become a popular technique to non-invasively manipulate brain rhythms via external, periodic stimulation. However, there is still debate regarding its underlying mechanisms and effects on brain activity. Here, we used EEG recordings during a visual entrainment paradigm to assess characteristic changes in the spectral content of EEG signals due to entrainment. Our results demonstrate that entrainment not only increases synchrony between neural oscillations and the entraining stimulus but also elicits previously unreported spectral tuning effects and long-lasting after-effects. These findings offer compelling evidence for the presence of dedicated, flexible, and adaptive mechanisms for neural entrainment, which may have key roles in adjusting the sensitivity and dynamic range of brain oscillators in response to environmental temporal structures.

**Supplementary Information:**

The online version contains supplementary material available at 10.1186/s12993-024-00259-6.

## Introduction

One of the methods that is becoming increasingly popular in cognitive neuroscience is the use of non-invasive brain stimulation techniques to induce targeted manipulations of neural rhythms [36, 46, 53, 74, 91, 92]. This method draws inspiration from phenomena such as the synchronized flashing of fireflies and the synchronization of pendulum clocks [13, 86, 97], where two or more oscillatory systems align their frequency to reach a state of unison [28, 68, 72]. It is believed that a very similar process occurs in the brain in response to periodic stimuli, such as rhythmic sounds or flashing lights [47, 58, 76, 91], with neural oscillators resetting and aligning their phase to the rhythm of the external stimulus.

This phenomenon, known as neural entrainment, is now recognized as a canonical mechanism in neural processing [35, 37, 44, 49, 51, 81], supporting functions such as speech processing [27, 48, 62, 82], temporal integration [6, 14, 77] and event anticipation [87, 99]. With the increasing popularity of this technique, more studies have focused on entraining brain activity at the alpha rhythm (8–13 Hz) to provide direct causal evidence of its proposed roles in modulating perception and attention [26, 33, 41, 46, 80], for relationship between alpha activity, perception and attention, see [15, 56, 57, 66, 67, 69, 71, 79]. The typical approach involves inducing entrainment at the target frequency (e.g., 10 Hz) with relatively long periodic stimulation before assessing perceptual performance [19, 29, 32, 42, 54, 83, 84].

Neural entrainment remains, nonetheless, a controversial topic. Recently, there has been debate over whether the observed synchrony between neural activity and an entraining stimulus truly reflects a state of entrainment [19, 22, 38, 60, 84, 99] or is simply a byproduct of evoked responses and transient resonance effects [12, 24, 25, 31, 34, 43, 61]. Trains of evoked responses to a periodic stimulus may indeed camouflage as synchrony with an external stimulus [12, 42, 43]. Resonance effects, akin to ripples from a rock thrown into water, can also lead to phenomena that resemble entrainment [35, 72]. However, there are aspects that have been traditionally considered as unique signatures of pure entrainment [50, 91]. These include the alignment of an oscillator’s intrinsic rhythm to the entraining stimulus by direct interaction, forward entrainment effects persisting beyond the duration of the entraining stimulus [63, 64, 78], and the emergence of phenomena—i.e., changes in neural states—that cannot be reduced to the simple sum of trains of evoked responses.

**Fig. 1:**
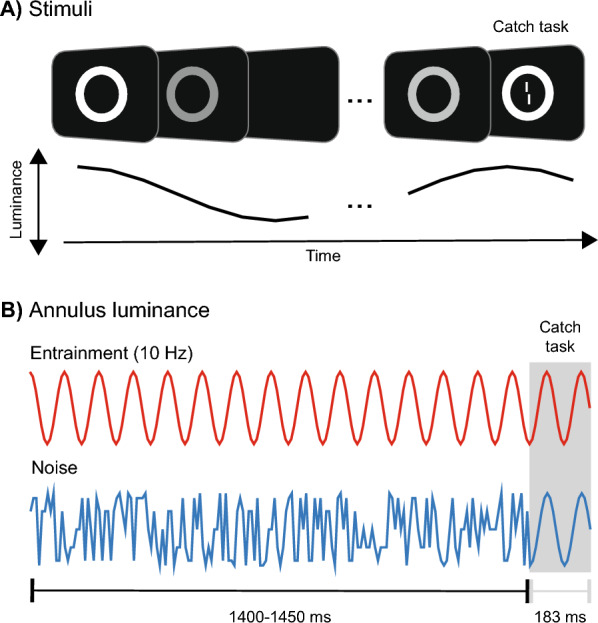
**A** Participants were exposed to a flickering annulus while asked to maintain their focus on the center, in order to perform a catch task at the end. **B** The annulus flickered at either 10 Hz or with noisy fluctuations. During the last 183 ms, the annulus flickered at 10 Hz in both conditions. Refer to “Methods” for additional details

We assessed these characteristics in an EEG experiment in which participants were exposed to a flickering annulus (Fig. 1). In two conditions, the luminance of the annulus varied according to either a 10 Hz regular rhythm or randomly, while participants focused on a secondary task at the annulus center. Both conditions ended with luminance oscillations at 10 Hz. We investigated (1) the effects of entrainment on EEG activity, specifically whether neural oscillators shifted their spectral properties towards the entraining rhythm, directly driven by this external stimulation; (2) forward entrainment effects outlasting the annulus stimulus; and (3) long-lasting effects, observable on the next trial, which would be indicative of a mechanism that transcends the simple sum of evoked responses within a single trial.

While we found evidence of both spectral changes and forward effects consistent with entrainment, confirming prior studies, our results also revealed novel aspects. Entrainment not only led to phase alignment and power modulations at the entrained frequency but also a marked decrease in power at neighbouring, non-stimulated frequencies. We termed this effect 'tuning', suggesting a mechanism that increases signal-to-noise ratio and communication at the specific channel of the entrained rhythm. Additionally, entrainment resulted in clear 'after-effects', a phenomenon unreported so far, causing a marked decrease in tuning around the previously entrained frequency when new stimulation occurred. Together, these findings are consistent with true entrainment effects that temporarily set the neural oscillators to flexible and adaptable states of functional synchrony with an external stimulus.

## Results

### Tuning of neural rhythms to the entrained frequency

During EEG recordings, participants were exposed to an annulus stimulus flickering at either 10 Hz (10 Hz condition) or with random luminance values (noise condition, see “Methods”, Fig. 1).

**Fig. 2:**
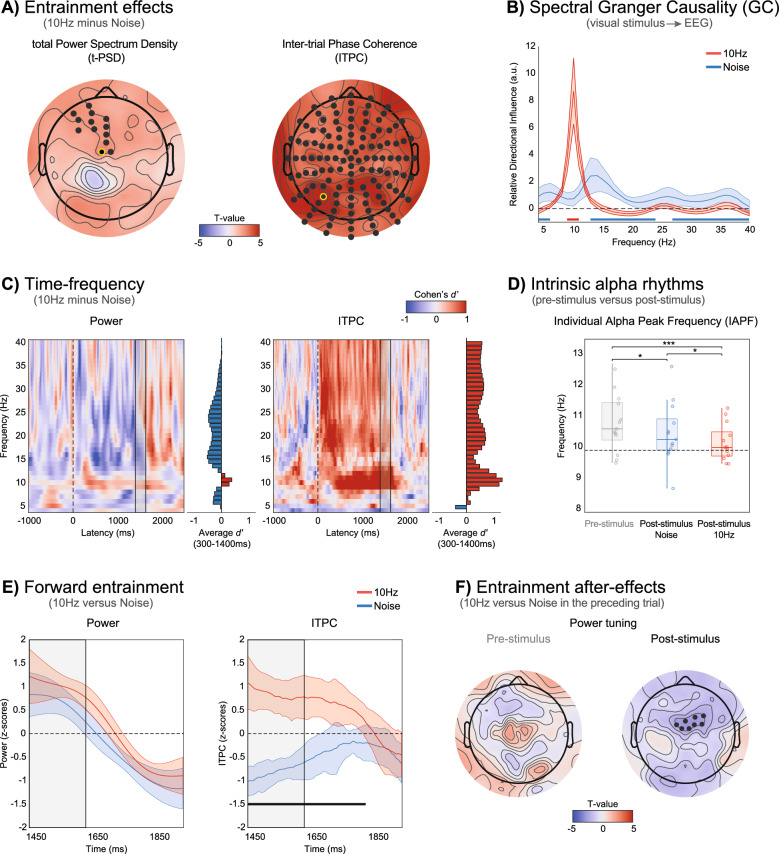
Testing key characteristics of neural entrainment. **A** Scalp analysis of entrainment effects in total PSD (t-PSD) and inter-trial phase coherence (ITPC). Electrodes showing significant post-stimulus differences between the 10 Hz entrainment and noise conditions are highlighted in black (cluster-based permutation test, *p* < 0.025, two-tailed). **B** Relative directional influence, which quantifies the directed influences from the stimulus luminance sequence to EEG activity in the 4–40 Hz range, was derived using spectral Granger causality (see “Methods” for details). Horizontal red and blue lines at the bottom of the plot indicate frequencies where the relative directional influence is higher for the 10 Hz and noise conditions, respectively. **C** Temporal dynamics of entrainment effects in power and ITPC, computed from the two electrodes showing the largest t-PSD and ITPC effects (respectively D1 and A8, surrounded in yellow in **A**). Effect sizes of the differences between the 10 Hz and noise conditions are shown for each frequency and time point. Histograms on the side of each plot represent the average effect size over the entire post-stimulus window (from 300 to 1400 ms). The dotted line marks the onset of the flickering annulus, while the gray rectangle indicates the time window containing the 10 Hz oscillating annulus in both conditions and the catch task. **D**. Individual alpha peak frequency (IAPF) estimated in the pre-stimulus (− 1000 to 0 ms, gray boxplot and dots) and post-stimulus window (300–1400 ms). Significant differences between 10 Hz (red boxplot and dots) and noise conditions (blue boxplot and dots) are highlighted with asteriks (*p < 0.05, ***p < 0.001). **E** Forward entrainment effects in power and ITPC. Power and ITPC values are z-scored within participants and across conditions. Significant differences between 10 Hz and noise conditions (respectively in red and blue, 95% CI) are indicated by the black line (i.e., only found in ITPC, *p* < 0.05, FDR corrected, Cohen's *d*′ = 2.7). The gray rectangle indicates the time window containing the 10 Hz oscillating annulus and the catch task in both conditions. **F** Scalp analysis of entrainment after-effects in t-PSD, normalized by neighboring frequencies (e.g., power tuning, see “Results” and “Methods”), and ITPC. Topographies represent the difference in power tuning and ITCP as a function of the condition on the preceding trial (10 Hz vs. noise), estimated separately for the current pre-stimulus or post-stimulus interval. Significant electrodes are highlighted in black (cluster-based permutation test, *p* < 0.025, two-tailed)

We first assessed entrainment effects in the power and phase of EEG activity at all electrodes, in a post-stimulus time window (from 300 to 1400 ms after the onset of the annulus). In the analysis of power, we estimated a measure of total power spectral density (t-PSD; see “Methods”), which includes both induced and evoked components [5, 88] at the entrained frequency (10 Hz). Cluster-based permutation statistics over the entire scalp revealed one significant cluster where 10 Hz activity increased in the 10 Hz condition compared to the Noise condition (*p* < 0.025, two-tailed, Cohen's *d*′ in the range 0.6–0.74 across channels in the significant cluster). This cluster was localized in a subset of anterior scalp electrodes (see Fig. 2A, first topography). In the analysis of phase, we computed the inter-trial phase coherence (ITPC; see “Methods”), quantifying the degree of phase alignment across trials, and compared ITPC values between conditions. Cluster-based permutation statistics revealed a strong and distributed increase in ITPC at 10 Hz in the 10 Hz condition at all electrodes (*p* < 0.025, two-tailed; Cohen's *d*′ in the range 0.69–1.68 across channels; see Fig. 2A, second topography). The significant clusters in the two analyses remained highly similar, regardless of the tested cluster-level α threshold (0.025 or 0.05; results in Fig. 2A are shown with cluster-level α = 0.025). Thus, both power and ITPC exhibited evident effects of entrainment, although ITPC effects were more pronounced and distributed. To verify that the changes in power and phase synchronization were supported and likely due to direct influences from the external rhythm to EEG signals, we conducted a pairwise spectral Granger causality analysis [10, 30] between the visual signals on each trial and the EEG activity at each electrode, separately for the two stimulation conditions. After averaging the obtained measure of relative directional influences across all electrodes (see “Methods”), the results revealed a significantly greater contribution of the external stimulus in driving 10 Hz EEG activity across the scalp during the entrainment condition, compared to the noise condition (Fig. 2B).

We then examined the temporal dynamics of entrainment effects using a time–frequency decomposition (Morlet wavelet convolution, 4–40 Hz, see “Methods”), focusing on electrodes exhibiting the most significant power and phase effects (D1 for t-PSD; A8 for ITPC). Effect sizes (Cohen’s *d*′) of the difference between conditions revealed that power increases due to entrainment, limited mostly to the entrained frequency, were also accompanied by a marked decrease in power at neighboring frequencies (e.g., above 12 and below 8 Hz, Fig. 2C, first time–frequency plot), an effect that we refer to as ‘tuning’. In a supplementary analysis, we assessed these tuning effects by normalizing t-PSD at 10 Hz using neighboring frequencies (5–6 and 14–15 Hz) and by directly comparing power changes in those neighboring bands. This analysis revealed additional effects localized at occipital electrodes (see Supplementary Material, Fig. S1).

Phase effects, on the other hand, were larger and evident throughout the entire entrainment period and beyond, with increased ITPC observed at 10 Hz and its harmonics (Fig. 2C, second time–frequency plot). Thus, entrainment effects in power and phase were largely dissociable.

Focusing on the effects on power, we investigated whether the tuning of EEG power observed around the entrained frequency was consistent with a shift in the dominant alpha peak of each individual (individual alpha peak frequency, IAPF; [45]). This being the case, the estimated IAPF during entrainment should differ from that found in pre-stimulus intervals and shifted towards the 10 Hz rhythm of the annulus. This was confirmed by a one-way repeated measures ANOVA (three levels: pre-stimulus IAPF, post-stimulus IAPF in the 10 Hz or Noise conditions), which revealed a significant main effect of condition (F(2, 12) = 8.96, *p* = 0.001; Fig. 2D), with a significant difference between the 10 Hz entrainment and both the pre-stimulus interval (W = 90, *p* < 0.001, Cohen's *d*′ = 0.8, Wilcoxon signed-rank test) and the Noise condition (W = 76, *p* = 0.03, Cohen's *d*′ = 0.4). IAPF values in the 10 Hz condition clustered around the entrained frequency, with no significant difference from 10 Hz (W = 58, *p* = 0.41, Cohen's *d*′ = 0.09; Fig. 2D).

### Forward entrainment effects

To investigate whether entrainment effects outlasted the duration of the annulus, we extracted time–frequency power and ITPC values at the entrained frequency. Power and ITPC were compared between the 10 Hz and noise conditions within a 500 ms time window starting 1450 ms after the onset of the annulus. This window covered the final presentation of the annulus along with the display of the central stimuli used in the catch task (lasting either 133 or 183 ms depending on the stimulation duration, i.e., 1400 or 1450 ms), and a subsequent blank interval. Note that, during the final presentation of the annulus, both conditions featured luminance oscillations at 10 Hz (Fig. 1B). Therefore, any forward entrainment effect persisting beyond the annulus presentation could not be due simply to evoked responses or resonance activity triggered by the final cycles of the annulus.

In the power analysis, no significant differences were observed between conditions at any of the time points considered (*p* > 0.05, paired t-test, after FDR correction; Fig. 2E, first plot). In contrast, ITPC exhibited a sustained increase in the 10 Hz condition that extended well beyond the end of the stimulation period (from 1450 to 1832 ms, encompassing 200 ms after the end of the central stimulus; *p* < 0.05, paired t-test, FDR correction, average Cohen’s *d*′ = 2.7; Fig. 2E, second plot).

### Long-lasting after-effects of entrainment

In this final analysis, we investigated whether the effects of entrainment persisted over subsequent trials, a result that would be indicative of a mechanism with long-lasting neural dynamics that extend well beyond the stimulation itself [16, 59, 90, 98]. We focused on the two primary effects found in the analyses above, the tuning of power spectrum and the modulation of ITPC.

We quantified tuning by normalizing t-PSD at 10 Hz by neighboring frequencies (see “Methods”) and compared the tuning observed in the current trial as a function of the stimulation condition (10 Hz entrainment or Noise) on the preceding trial. This analysis was conducted separately for the pre-stimulus interval (− 1000 to 0 ms) and the post-stimulus interval during the current trial (300–1400 ms) to disentangle effects manifesting on ongoing activity or during responses to new stimulation. No changes in tuning due to the preceding trial condition were observed in the power tuning when considering the pre-stimulus interval (*p* > 0.025; cluster-based permutation statistics, Fig. 2F, pre-stimulus topographies). However, in the post-stimulus window, a significant cluster of fronto-central electrodes showed decreased tuning after entrainment compared to the Noise condition (*p* < 0.025, Cohen's *d*′ in the range 0.61–0.73 across channels in the significant cluster, cluster-based permutation statistics; Fig. 2F, post-stimulus topography). This cluster appeared relatively localized, as it was significant only at the cluster-level α threshold of 0.025 and not at 0.05. In addition, no after-effects were found when considering the raw t-PSD at 10 Hz or differences at neighboring frequencies (5–6 Hz or 14–15 Hz, all *p* > 0.025; cluster-based permutation statistics). Similarly, for ITPC at 10 Hz, no significant after-effects were observed in either the pre- or post-stimulus intervals (*p* > 0.025; cluster-based permutation statistics). These results suggest subtle yet robust after-effects that are evident exclusively when contrasting power at the frequency entrained on the previous trial with its neighboring frequency bands.

## Discussion

We assessed key characteristics of neural entrainment in an EEG experiment involving periodic visual stimulation at the alpha rhythm (10 Hz). We compared a condition with 10 Hz entrainment with a condition involving a non-periodic stimulation. Our findings extend beyond the typical effects of increased power and synchronization at the entrained frequency, revealing several key observations:

1. Frequency shift and tuning: we observed a shift in the dominant alpha frequency, characterized by increased power at the entrained rhythm and decreased power in neighboring frequencies. This tuning was accompanied by widespread phase synchronization with the entraining rhythm and directed spectral influences at the entrained frequency from the periodic stimulus to brain activity.
2. Forward entrainment: we showed a persistence of synchronization effects (but not power effects) after the removal of the entraining rhythm.
3. Long-lasting after-effects: we identified long-lasting after-effects of tuning, similar to neural adaptation, which were observable in trials following the 10 Hz entrainment.

Previous studies have questioned whether entrainment effects could be explained by simpler mechanisms, such as the superimposition of neural responses to periodic stimuli or resonance phenomena [72]. Superimposition leads to trains of evoked responses that add a periodic component to the EEG spectrum [12, 42, 43]. Resonance results in reverberating periodic responses that also add to the ongoing EEG spectrum [35]. Both assume effects that are specific to the frequency of the entraining stimulus (and its harmonics).

While these mechanisms can, in principle, explain synchronization [12], and even forward entrainment by assuming that neural activity continues to reverberate for several cycles after stimulus removal [35, 43], they fail to provide a straightforward explanation for the tuning and long-lasting after-effects observed in our study. Neither of them can indeed explain the drastic reduction in power at frequencies outside the entrained one. Even more, neither of these alternative mechanisms can, in no way, explain after-effects. Hence, while we do not exclude that both superimposition and resonance can be at play, during periodic stimulation, the effects of entrainment characterized here appear strongly suggestive of dedicated mechanisms, whose behavior is more than the sum of evoked and resonating responses.

Our findings suggest that periodic stimulation interacts with endogenous rhythms rather than simply superimposing or resonating with them. This is supported by the observed shift in IAPF towards the entrained 10 Hz rhythm (Fig. 2D). In the 10 Hz condition, this shift, evident when comparing pre- and post-stimulus IAPF, exceeded the effects seen in the Noise condition. Thus, it is plausible that the intrinsic alpha rhythm adjusted its frequency to align with the external entraining rhythm, a hallmark feature of neural entrainment [43, 84].

Our results also revealed dissociable effects of entrainment on spectral features. Strong phase effects were largely specific to the entrained frequency and its harmonics, consistently found across all electrodes and persisting beyond the duration of the stimulus. This widespread phase locking was likely driven by the direct influence of the 10 Hz entraining stimulus on global EEG activity, as revealed by spectral GC analysis (Fig. 2B). This suggests that the external periodic stimulus exerts a strong drive on neural activity, eventually promoting phase synchronization across widespread brain areas.

In contrast, power effects were observed only during stimulation, with an increase at the entrained frequency and a reduction across neighboring frequencies. Specifically, when analyzing raw t-PSD, entrainment effects—namely, increases in power at 10 Hz—were detectable in a subset of fronto-central electrodes. However, time–frequency analysis revealed that, as the entraining stimulation progressed, the effects were not only marked by increased power at 10 Hz but also by significant decreases at adjacent frequencies (Fig. 2C). Similar patterns have been observed before [84].

To better assess these effects, which we refer to as spectral tuning, we normalized t-PSD using neighboring frequencies and, additionally, performed a direct comparison of power effects within these neighboring bands (see Supplementary Material). This approach revealed effects also at occipital electrodes (Fig. S1). That is, the normalized t-PSD showed power effects not only in fronto-central electrodes but also across a distributed set of occipital electrodes. The comparison of power effects at nearby frequencies also showed decreases at 5–6 Hz and 14–15 Hz during 10 Hz entrainment, predominantly localized to the posterior occipital electrodes. We propose that evaluating entrainment effects by contrasting neural activity at the entrained frequency with that at neighboring frequencies provides several advantages: (1) enhanced sensitivity for precisely identifying how entrainment shapes the frequency spectrum; (2) reduced variability at the inter-individual level; and (3) improved detection of subtle tuning effects that may be overlooked in raw power analyses.

We speculate that these tuning effects may reflect neural dynamics that emerge specifically during periodic stimulation, whose role is to optimize neural sensitivity and dynamic range in response to periodic input. The topographies of these effects (Fig. S1) suggest that spectral tuning is mediated by widespread changes, likely involving both bottom-up and top-down mechanisms. This could account for the involvement of occipital clusters, as well as the more fronto-central ones.

After the end of the stimulation, we found forward entrainment effects that cannot be simply explained by resonance phenomena. In both the 10 Hz and noise conditions, the final part of the stimulation sequence included a 10 Hz periodic luminance modulation (Fig. 1). This could have produced comparable reverberating responses in both conditions. However, we found phase synchronization effects that persisted for several hundred milliseconds only after the 10 Hz entrainment condition, indicating that these effects were driven by prolonged entrainment at 10 Hz prior to the final stimulation sequence. Importantly, while forward entrainment effects were evident in phase synchronization, we did not observe sustained power increases, even though there was a qualitative trend (Fig. 2E). This contrasts with previous findings [64, 84]. It is possible that differences between our paradigm and previous research may explain the lack of clear forward entrainment effects on power. For example, the presence of 10 Hz stimulation in the final part of both conditions, as well as our data-driven approach to examine power effects in a cluster of fronto-central electrodes (Fig. 2A), might explain this discrepancy, given that earlier studies typically reported forward entrainment effects in power primarily at occipital electrodes. Further research is needed to clarify these aspects.

To the best of our knowledge, this study is the first to report an after-effect in neural entrainment induced by visual stimulation. Previous studies using transcranial alternating current stimulation (tACS) have observed long-lasting effects, where power increases at the entrained frequency persist for several minutes following prolonged stimulation (e.g., 20 min; [40, 59, 96]). These sustained effects have been attributed to spike-timing-dependent plasticity, which selectively modulates synaptic connections based on the resonance frequencies of the neural circuits involved [98]. In contrast, our findings reveal that even short periods of visual entrainment (lasting only a few seconds) can induce negative after-effects, wherein the entrainment effects reverse post-stimulation. Rather than a persistent increase in power at the entrained frequency, we observed a reduction in power following trials with entrainment (Fig. 2F), suggesting a compensatory rebound mechanism. This phenomenon may reflect a short-term form of neural adaptation or habituation processes [16, 17, 75, 90, 93, 94], where the brain transiently suppresses activity at the previously entrained frequency to restore baseline oscillatory dynamics. Within this view, evidence of after-effects appears as the ultimate proof of a flexible and efficient mechanism for neural entrainment that adjusts to the prevailing rhythms of the environment, increasing sensitivity to different rhythms after repetitive exposure to the same one.

After-effects associated with power tuning were localized in a restricted set of fronto-central electrodes, suggesting the involvement of higher-order brain areas, beyond early visual cortex. One possibility is that the mechanisms engaged by entrainment operate at the level of frontal circuits involved in encoding temporal structures for attention [8, 11, 18]. It must be noted, however, that in our paradigm the entraining stimulus—the annulus—required no attention and was completely task irrelevant. Thus, such mechanisms must operate in a rather automatic and task-independent fashion.

Several key questions remain open for future research. First, since the behavioral task was only incidental in this study, we could not directly assess the effects and aftereffects of entrainment on visual processing. Second, we focused exclusively on the alpha rhythm; future studies are needed to determine whether similar characteristics of entrainment apply to periodic stimulation at other frequencies. Third, a further direct test of pure entrainment could involve adjusting the luminance of the driving stimulus to evaluate whether stronger stimulation leads to stronger synchronization. Lastly, while we observed a global shift in the alpha peak frequency toward the entrained rhythm, future work should assess the spatial distribution of these effects, to verify the involvement of shifts from different endogenous frequencies, such as theta in frontal regions or alpha in occipital areas.

Although the exact mechanisms and the neural sources underlying the entrainment effects reported here remain speculative, we believe that our work paves the way for future investigations. For instance, future studies may investigate tuning effects targeting individual alpha peaks and broader frequency ranges, assessing their canonical nature and relation to the intrinsic dominant frequency of an individual brain. Likewise, further research may explore the interplay between tuning, after-effects, and performance in perceptual and cognitive tasks using tailored experimental designs, given that the task employed here primarily served as a means to ensure focus on the screen.

## Conclusions

In sum, our findings reveal important characteristics of neural entrainment that support the existence of dedicated, flexible and adaptive mechanisms in the brain. These findings are of key relevance for ongoing debates surrounding the nature of entrainment and considering the increasing use of these techniques to investigate the causal role of brain rhythms in perception and cognition.

## Methods

### Participants

Fourteen right-handed healthy adults (age range: 20–27 years old; 6 females) with no psychiatric or neurological history participated in the experiment as volunteers or for monetary reward (30 CHF per hour). Participants had normal or corrected to normal vision (Freiburg acuity test, threshold for inclusion: > 1; [3]) and received instructions and written informed consent before the experiment. The experiment was conducted in accordance with the local ethics committee and complied with the Declaration of Helsinki.

### Apparatus

The study was conducted at the Laboratory of Psychophysics (LPSY) at the École Polytechnique Fédérale de Lausanne (EPFL). Participants were seated in a dimly lit, electromagnetic shielded room in front of an ASUS VG248QE LCD monitor (1920 × 1080 pixels, 24.5″ screen size, 120 Hz refresh rate), sitting at a distance of 100 cm. Stimuli were presented via custom-made scripts written in MATLAB R2022b (MathWorks) and the Psychophysics Toolbox [9].

### Stimuli and procedure

A trial example is depicted in Fig. 1. Participants were instructed to fixate on the center of a black screen (1 cd/m^2^) for the entire trial. Each trial started with the presentation of a flickering annulus around the screen center (diameter = 10°, oval frame thickness in pixels = 50; Fig. 1A). Two conditions alternated randomly (Fig. 1B). In the 10 Hz condition, the annulus luminance fluctuated in the 1–50 cd/m^2^ range with a sinusoidal modulation at a constant frequency of 10 Hz. The starting phase was always the same, beginning at maximal luminance. In the *Noise* condition, luminance values mirrored those in the 10 Hz condition but were temporally shuffled, producing no fixed frequency—i.e., resembling white noise. In both conditions, the annulus was presented for either 1400 or 1450 ms and was always followed by an additional 183 ms period of rhythmic fluctuations at 10 Hz (Fig. 1B).

During this last 183 ms, participants were also presented with a visual task, involving a Sequential Metacontrast Paradigm (SQM; [65]), shown at the annulus center. The first central stimulus consisted of a vernier offset (length = 1600 arcsec, width = 70 arcsec, vertical gap = 120 arcsec) followed by a sequence of five pairs of flanking lines (for further details on the SQM, which is typically used to investigate long-lasting feature integration; see [23, 39]). Participants were instructed to attend to the right stream and report the perceived offset. We chose the SQM to assess its suitability as a perceptual task in entrainment protocols for a separate project, which also concerned the presentation of one or two opposite verniers in the stream (i.e., for 6 participants, the 3rd line in the attended stream was also offset, always in the opposite direction compared to the central vernier offset) and the choice of different entrainment durations (i.e., 1400 vs 1450 ms; both aspects not considered for the purpose of the current study analyses). Thus, the central task here was considered accessory and served as a ‘catch’ task to ensure participants' attention remained focused on the center, while also confirming the task's feasibility. The size of each vernier offset was calibrated for each participant prior to the main experiment in order to achieve 75% discrimination accuracy when presented alone in the stream. Performance in vernier offset discrimination remained similar across conditions (10 Hz vs. noise): around 75% of correct responses when only the central vernier was presented, and around 50% when two opposite verniers were shown, as they cancel each other out [23].

Following the participant responses, the next trial was preceded by a fixed 1-s blank interval, plus an additional randomized interval ranging from 0.4 to 1.1 s in 0.1-s increments. This ensured that, in both 10 Hz and noise conditions, the onset of the annulus remained difficult to predict accurately.

In total, participants completed 400 trials, organized into 10 blocks of 40 trials each. The conditions (10 Hz vs. noise) were randomized within each block. Participants initiated each block by pressing a button.

### Electroencephalography recordings and preprocessing

EEG data acquisition was performed using a 128-channel ActiveTwo EEG system (Biosemi, Amsterdam, The Netherlands), with a sampling rate of 2048 Hz. The recorded EEG signals were downsampled to 250 Hz using an anti-aliasing filter (0.9 Hz cutoff frequency, 0.2 Hz transition bandwidth) and detrended according to the PREP plugin detrending procedure [7]. Then, data were low-pass filtered at 40 Hz and epoched from − 1 to 2.5 s relative to the onset of the annulus. Bad epochs and channels were identified via visual inspection and removed. Additionally, signal components related to eye and muscular artifacts were identified after ICA decomposition (Picard plugin; [1, 2]) with a semi-automatic procedure based on ICLabel bad components detection [73] and further visual assessment. Finally, removed channels were interpolated using a spherical spline interpolation [70], and a re-referencing to the common average reference was applied to the signals [52]. EEG data were preprocessed with EEGLAB and its plugins (version v2022.1; [20, 21]) in MATLAB (The MathWorks Inc., Natick, USA). In total, 4.85% of the electrodes were interpolated, while 6.35% of the epochs and 12.07% of the independent components were removed.

### EEG analysis of entrainment

Our analyses focused on testing three main effects of entraining EEG activity, comparing the 10 Hz and the Noise conditions. The target effects and methods used are described below.

#### Entrainment effects on ongoing neural oscillations

We sought to identify changes in the intrinsic properties of neural oscillations, specifically power, phase, and frequency content, resulting from entrainment at 10 Hz. Theses analyses were conducted within a time window from 300 to 1400 ms after stimulus onset, i.e., excluding the initial sequence of evoked responses to the stimulus onset, as well as the final sequence involving the SQM stimuli and the 10 Hz oscillating annulus in both conditions.

We compared the total power at the entrained frequency (10 Hz) between the 10 Hz and noise conditions. Total power spectral density (t-PSD) estimates within the 4–40 Hz frequency range were obtained for each channel and condition using a 1024-point fast Fourier transform (*fft*(), MATLAB R2022b). t-PSD considers both evoked and induced components by averaging single-trial PSD estimates, unlike other methods that directly apply frequency decompositions on the trial average [5, 88].

In the analysis of phase effects, we compared the phase alignment across trials with the entrained frequency between 10 Hz and noise conditions. Phase alignment was evaluated through inter-trial phase coherence (ITPC; *newtimef*() in EEGLAB, with wavelet cycles = 7; [89, 95]).

Differences between conditions in t-PSD and ITPC, were assessed at the scalp level, via non-parametric cluster-based permutation statistics (paired t-test with α = 0.025, two-tailed, number of permutations = 10,000). In addition, two different cluster α values were tested (cluster-level α = 0.05 and 0.025), determining the critical value used for thresholding the sample-specific T-statistics (i.e., the size of the original clusters entering the permutation).

To inspect the temporal dynamics of these entrainment effects, we then applied a time–frequency analysis, estimating power and ITPC after Morlet wavelet convolution in the 4–40 Hz range (37 linearly spaced frequency, wavelet cycles = 7). These analyses were performed on the electrodes showing the larges t-PSD (D1) and ITPC (A8) effects, and were represented as effect sizes (Cohen’s *d*′ of the difference 10 Hz minus noise condition; Fig. 2C).

We also assessed spectral Granger causality (GC) between neural oscillatory activity (downsampled at the monitor frame rate, 120 Hz) and the external visual stimulus under the two experimental conditions. GC was estimated for each participant, focusing on the causal influence of the external stimulus on neural oscillators (stimulus to EEG, 'in') and the reverse direction (EEG to stimulus, 'out'). The MVGC toolbox [4] was used for the analysis, applying the functions *tsdata_to_var*() to fit a vector autoregressive model and *var_to_autocov*() to compute the spectral GC using *autocov_to_spwcgc*(). The model order was set to 10 lags.

This analysis involved estimating pairwise spectral GC between the stimulus and activity in each EEG channel, followed by averaging the results across channels. To address the potential issue of singular and ill-defined covariance matrices in GC estimation—caused by the noiseless 10 Hz stimulus signal leading to rank deficiency and numerical instability—we randomly perturbed the periodic luminance signal in entrainment trials with Gaussian noise, using a standard deviation of 0.75 times the maximum signal value (different proportions were tested, yielding consistent results).

For each participant, we calculated a measure of relative directional influence as (in–out)/out, providing a normalized metric of asymmetry between the influence from the stimulus to the EEG activity and the reverse direction. This measure evaluates the extent to which the stimulus drives neural oscillators compared to the influence of EEG activity on the stimulus. Relative directional influences were assessed across the frequency range of 4–40 Hz. Differences between the two conditions (entrainment vs. noise) were tested using paired t-tests, with p-values corrected for multiple comparisons using the false discovery rate method (FDR; [85]).

Next, we focused on the frequency content. Specifically, we aimed to test whether entrainment results in a shift in the dominant alpha frequency of each individual brain. To this aim, we first estimated the individual alpha peak frequency using pre-stimulation EEG activity (− 1000 to 0 ms). This involved calculating t-PSD within the 1–40 Hz frequency range and fitting a linear model to the logarithm of the frequency axis versus the average power. By obtaining the residuals of this linear fit, we effectively removed the 1/f component from the PSD and identified the peak within the alpha frequency range (8–13 Hz) for each participant [57]. We repeated this procedure to estimate IAPF in post-stimulus activity (from 300 to 1400 ms), separately for both conditions (10 Hz and noise). One participant was excluded from this analysis as no clear alpha peak was found during stimulation in both 10 Hz and noise conditions. The estimated IAPF values obtained during the pre-stimulation period, as well as during the 10 Hz and noise conditions, were then subjected to a one-way repeated measures ANOVA and post-hoc Wilcoxon signed-rank tests.

#### Forward entrainment

We tested forward entrainment—i.e., the persistence of entrainment effects after stimulation, by comparing the power and ITPC at 10 Hz, obtained via time–frequency decomposition, between the 10 Hz and noise conditions in a 500 ms time window starting at 1450 ms from the annulus onset (depending on the duration of the annulus presentation, i.e., 1400 or 1450 ms, this window encompassed either 133 or 183 ms of 10 Hz stimulation and, respectively, 367 or 317 ms of blank screen). Statistical comparison relied on paired t-test (α = 0.05), with p-values corrected for multiple comparisons using FDR.

#### Entrainment after-effects

We investigated persistent effects of entrainment on subsequent trials (after-effects) by comparing power effects and ITPC after trials with 10 Hz or noise. In evaluating after-effects, we also considered pre-stimulus (− 1000 to 0 ms before the onset of the annulus in the current trial), in addition to post-stimulus effects (300 to 1400 ms after the onset of the annulus). Based on the observed ‘tuning’ effects (see “Tuning of neural rhythms to the entrained frequency”), we here used t-PSD at the entrained frequency (10 Hz), normalized by neighboring frequencies (5–6 Hz and 14–15 Hz; [55]). Statistical assessment followed the same cluster-based approach at the scale level described above.

## Supplementary Information

Supplementary material 1.

## Data Availability

The datasets generated and/or analysed during the current study are available in the Open Science Framework repository (osf.io/qz7mx).

## Abbreviations

EEG: Electroencephalogram
FDR: False discovery rate
GC: Granger causality
IAPF: Individual alpha peak frequency
ITPC: Inter-trial phase coherence
SQM: Sequential metacontrast paradigm
(t-)PSD: (Total) power spectrum density
tACS: Transcranial alternating current stimulation (tACS)
