# Correction: Spectral tuning and after‑effects in neural entrainment

**DOI:** 10.1186/s12993-025-00295-w

**Published:** 2025-09-19

**Authors:** Maëlan Q. Menetrey, David Pascucci

**Affiliations:** 1 Laboratory of Psychophysics, Brain Mind Institute, Ecole Polytechnique Federale de Lausanne (EPFL), Lausanne, Switzerland; 2 Psychophysics and Neural Dynamics Lab, Department of Radiology, Lausanne University Hospital (CHUV) and University of Lausanne (UNIL), Lausanne, Switzerland; 3 The Sense Innovation and Research Center, Lausanne, Switzerland

**Correction: Behavioral and Brain Functions (2024) 20:29** 10.1186/s12993-024-00259-6

In the original publication of this article [1], there was an error in Fig. 2. In panels 2A, 2C, and 2F, the value “0” was misaligned and should have appeared at the center of the color bars. For completeness and transparency, both the incorrect and corrected versions of Fig. 2 are displayed below. The original article has been corrected.

Incorrect Figure 2:

**Fig. 2:**
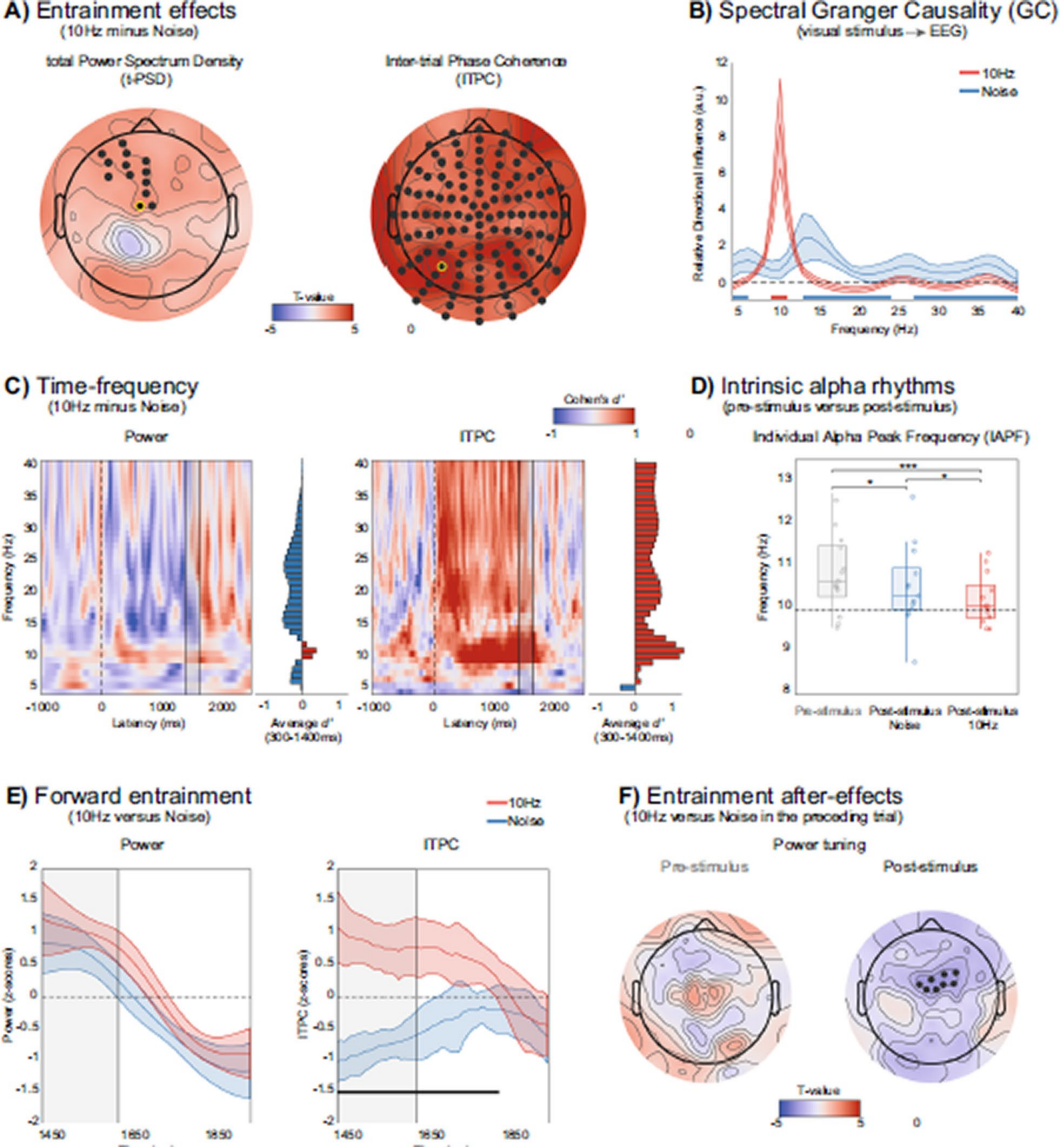
Testing key characteristics of neural entrainment. **A** Scalp analysis of entrainment effects in total PSD (t-PSD) and inter-trial phase coherence (ITPC). Electrodes showing significant post-stimulus differences between the 10 Hz entrainment and noise conditions are highlighted in black (cluster-based permutation test, *p* < 0.025, two-tailed). **B** Relative directional influence, which quantifies the directed influences from the stimulus luminance sequence to EEG activity in the 4–40 Hz range, was derived using spectral Granger causality (see “Methods” for details). Horizontal red and blue lines at the bottom of the plot indicate frequencies where the relative directional influence is higher for the 10 Hz and noise conditions, respectively. **C** Temporal dynamics of entrainment effects in power and ITPC, computed from the two electrodes showing the largest t-PSD and ITPC effects (respectively D1 and A8, surrounded in yellow in **A**). Effect sizes of the differences between the 10 Hz and noise conditions are shown for each frequency and time point. Histograms on the side of each plot represent the average effect size over the entire post-stimulus window (from 300 to 1400 ms). The dotted line marks the onset of the flickering annulus, while the gray rectangle indicates the time window containing the 10 Hz oscillating annulus in both conditions and the catch task. **D** Individual alpha peak frequency (IAPF) estimated in the pre-stimulus (− 1000 to 0 ms, gray boxplot and dots) and post-stimulus window (300–1400 ms). Significant differences between 10 Hz (red boxplot and dots) and noise conditions (blue boxplot and dots) are highlighted with asteriks (*p < 0.05, ***p < 0.001). **E** Forward entrainment effects in power and ITPC. Power and ITPC values are z-scored within participants and across conditions. Significant differences between 10 Hz and noise conditions (respectively in red and blue, 95% CI) are indicated by the black line (i.e., only found in ITPC, *p* < 0.05, FDR corrected, Cohen’s *d*′ = 2.7). The gray rectangle indicates the time window containing the 10 Hz oscillating annulus and the catch task in both conditions. **F** Scalp analysis of entrainment after-effects in t-PSD, normalized by neighboring frequencies (e.g., power tuning, see “Results” and “Methods”), and ITPC. Topographies represent the difference in power tuning and ITCP as a function of the condition on the preceding trial (10 Hz vs. noise), estimated separately for the current pre-stimulus or post-stimulus interval. Significant electrodes are highlighted in black (cluster-based permutation test, *p* < 0.025, two-tailed)

Corrected Fig. 2:

**Fig. 2:**
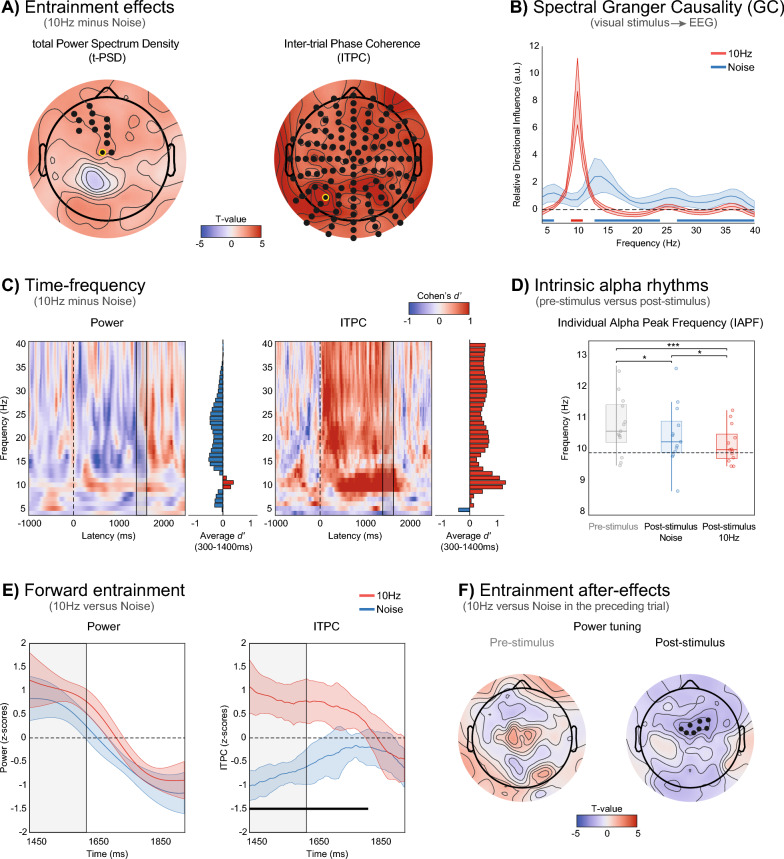
Testing key characteristics of neural entrainment. **A** Scalp analysis of entrainment effects in total PSD (t-PSD) and inter-trial phase coherence (ITPC). Electrodes showing significant post-stimulus differences between the 10 Hz entrainment and noise conditions are highlighted in black (cluster-based permutation test, *p* < 0.025, two-tailed). **B** Relative directional influence, which quantifies the directed influences from the stimulus luminance sequence to EEG activity in the 4–40 Hz range, was derived using spectral Granger causality (see “Methods” for details). Horizontal red and blue lines at the bottom of the plot indicate frequencies where the relative directional influence is higher for the 10 Hz and noise conditions, respectively. **C** Temporal dynamics of entrainment effects in power and ITPC, computed from the two electrodes showing the largest t-PSD and ITPC effects (respectively D1 and A8, surrounded in yellow in **A**). Effect sizes of the differences between the 10 Hz and noise conditions are shown for each frequency and time point. Histograms on the side of each plot represent the average effect size over the entire post-stimulus window (from 300 to 1400 ms). The dotted line marks the onset of the flickering annulus, while the gray rectangle indicates the time window containing the 10 Hz oscillating annulus in both conditions and the catch task. **D** Individual alpha peak frequency (IAPF) estimated in the pre-stimulus (− 1000 to 0 ms, gray boxplot and dots) and post-stimulus window (300–1400 ms). Significant differences between 10 Hz (red boxplot and dots) and noise conditions (blue boxplot and dots) are highlighted with asteriks (*p < 0.05, ***p < 0.001). **E** Forward entrainment effects in power and ITPC. Power and ITPC values are z-scored within participants and across conditions. Significant differences between 10 Hz and noise conditions (respectively in red and blue, 95% CI) are indicated by the black line (i.e., only found in ITPC, *p* < 0.05, FDR corrected, Cohen’s *d*′ = 2.7). The gray rectangle indicates the time window containing the 10 Hz oscillating annulus and the catch task in both conditions. **F** Scalp analysis of entrainment after-effects in t-PSD, normalized by neighboring frequencies (e.g., power tuning, see “Results” and “Methods”), and ITPC. Topographies represent the difference in power tuning and ITCP as a function of the condition on the preceding trial (10 Hz vs. noise), estimated separately for the current pre-stimulus or post-stimulus interval. Significant electrodes are highlighted in black (cluster-based permutation test, *p* < 0.025, two-tailed)
