# Supplementary material for: Spectral tuning and after-effects in neural entrainment
Source: Behav Brain Funct. 2024 Nov 21;20:29. doi: 10.1186/s12993-024-00259-6 (PMC11580347; doi:10.1186/s12993-024-00259-6)
Supplement: Supplementary file 1 — Supplementary material 1. [file 12993_2024_259_MOESM1_ESM.docx]

## Supplementary Material

*Spectral tuning in neural entrainment*

In our main analysis, we found entrainment effects when comparing the 10 Hz and Noise conditions in total power spectral density (t-PSD; see Methods), at the entrained frequency (10 Hz). This involved an increase in 10 Hz power during entrainment, observed in a subset of anterior scalp electrodes (Figure 2A, first topography). However, the time-frequency analysis across a broader range of frequencies revealed additional patterns (Figure 2C). Specifically, the increase in 10 Hz power was accompanied by a decrease in power at neighboring frequencies, with the strongest reductions occurring around 5 Hz and 15 Hz during 10 Hz stimulation. We refer to this effect as ‘spectral tuning’.

In this supplementary analysis, we examined the spatial distribution of these tuning effects across the scalp. First, we evaluated whether a normalized measure of power effects (Meigen & Bach, 1999), similar to that used in the after-effect analysis, could reveal a different topography compared to the raw t-PSD analysis. The rationale is that normalization by neighboring frequencies can help to identify tuning effects by providing a clearer contrast between the frequency being entrained (e.g., 10 Hz) and the surrounding frequencies.

Second, we directly assessed tuning effects at neighboring frequencies by comparing t-PSD in the 5-6 Hz and 14-15 Hz ranges between conditions. Both analyses employed non-parametric cluster-based permutation statistics (cluster-level α = .025; paired t-test with α = .025, two-tailed; number of permutations = 10’000).

The first analysis, focusing on normalized t-PSD at 10 Hz, revealed two significant clusters in fronto-central and occipital electrodes (*p* < .025, two-tailed; Cohen's *d’* ranging from 0.62 to 0.99 across channels; Figure S1A).

The second analysis, which examined tuning at neighboring frequencies, revealed significant decreases in both the 5-6 Hz and 14-15 Hz bands, localized to occipital electrodes. The effect at 5-6 Hz was highly localized (Cohen's *d’* ranging from 0.63 to 1.29 across channels; Figure S1B), while the effect at 14-15 Hz was more widespread (Cohen's *d’* ranging from 0.63 to 1.44 across channels; Figure S1C).

Overall, these findings suggest that tuning effects are more clearly localized to occipital electrodes when compared to the raw t-PSD analysis. Thus, normalizing by and assessing tuning at neighboring frequencies may offer a more sensitive measure than raw power alone, enhancing the detection of entrainment effects in both occipital and fronto-central regions.

**Figure S1. Tuning effects of entrainment.** A) Scalp analysis of entrainment effects at 10 Hz using t-PSD normalized by neighboring frequencies (5-6 Hz and 14-15 Hz). B-C) Tuning effects in the two neighboring frequency bands: 5-6 Hz (B) and 14-15 Hz (C). Electrodes showing significant post-stimulus differences between the 10 Hz entrainment and Noise conditions are highlighted in black (cluster-based permutation test, *p* < .025, two-tailed).
